# Our New York Letter

**Published:** 1891-05

**Authors:** 


### OUR NEW YORK LETTER.

NEW YORK, April 15, 1891.

La grippe has again made its appearance in this city, and this time with greatly renewed virulence. Last year, when it became known for the first time here, it was generally spoken of in a rather facetious manner, and people regarded it lightly; but at the present moment, when it is raging in all its fiercest intensity, striking down the strong, the vigorous and the hopeful, this disease is at the present time looked upon in a very serious light. Its pathology is obscure, and its treatment absolutely expectant. The deaths from this disease during the past few days have been so numerous, both in this city and Brooklyn, that undertakers have had more work than they have been able to attend to. One peculiar characteristic of the affection seems to be that the robust and apparently healthy succumb to its effects equally with the feeble and ill-nourished. It seems to bring out, in the individual afflicted with the malady, diseases which were heretofore harmless and gave him no trouble, and the complicating pneumonia, which, in a large majority of cases, accompanies it, is very difficult to treat satisfactorily. The type of the pneumonia these patients develop is characterized by an excess of bronchitis, and with an evident inability of the right heart to force the blood through the lungs. As a result of this condition of affairs, the patient soon begins to have sibilant and sonorous breathing over both lungs, often coarse rales, and then subcrepitant rales, with little or no expectoration. The bronchi, as a result of this, become filled up with mucus, and the inability of the right heart to propel the blood through the lungs, shows itself in the general venous congestion. Owing to this combination of feeble heart and general venous congestion, the patient goes from bad to worse until he finally succumbs to the malady. Death generally takes place in from twenty-four hours to three days after the onset of the pneumonia.

Another characteristic feature of this disease is that the means of treatment ordinarily applied for these complications are of such little avail here. Under ordinary circumstances a general bronchitis can be materially relieved by efficient cupping; but in this complicating bronchitis cupping does no good whatever. In the ordinary combination of venous congestion and feeble heart-action, hypodermic injections of nitroglycerine are found of service; in many of these cases this remedy is of no avail.

The only thing, therefore, that patients with grippe can do is to go to bed on the first appearance of the characteristic symptoms, which are high fever, frontal headache and marked general prostration, and remain in bed till these symptoms have entirely disappeared.

It may be of interest to your readers to know the result of the treatment that has been used in the case of Prof. Wyeth's patient with aneurism of the aorta, mentioned in the last issue of your journal. The operation has been in every way successful so far as coagulation of blood in thə sac was concerned. The patient was seen yesterday by the writer, and the aneurismal tumor had given place to a hard and solid mass filled apparently with coagulated blood fibrine. His respiration was normal, his appetite excellent, and he stated that he never felt so well in his life. He will soon be in a position to leave the hospital entirely cured of his trouble.

This procedure, the insertion of sterilized needles into the cavity of the aneurismal sac, as practiced by Dr. Wyeth in this case, is known as that of Maceuresi, of Glasgow, and the apparent safety and ease with which it was carried out will no doubt recommend its application in similar cases of aneurism.

Dr. H. Marion Sims, professor of gynecology at the New York Polyclinic, recently presented a patient with the following highly interesting condition: She was a woman, 39 years of age, and had been married fifteen years. She had had five children, the last one being born eleven months ago. On making a digital examination per vaginum, the doctor found an abnormal atrophy of the uterus, with slight laceration of the cervix. The woman was in every way well developed, physically, and had ceased to menstruate several months previously. The treatment consisted in the insertion into the cervical canal of a plain, hard rubber stem, the same as is applied in cases of dysmenorrhœa or hyperplasia of the uterus. It is a curious fact in practical therapeutics, that this same process, which is so efficacious in cases of dysmenorrhœa or uterine hyperplasia, has the very opposite effect in atrophy of the uterine body. The uterus of this patient, when she first presented at the clinic, was no larger than that of a girl ten or eleven years of age, but she had worn this stem but for a brief period when the uterus began gradually to reach its normal stage of development, and the woman commenced again to menstruate regularly as before.

A few words of caution are necessary to patients who wear a stem like this. They should be given instruction to come to the physician's office again, within a reasonable time, so as to ascertain whether the instrument is producing any irritable action on the tubes and ovaries or not, a condition which is apt to happen in a certain class of cases.

In speaking of the diagnosis of atrophy of the uterus, Dr. Sims did not consider it necessary to introduce a sound into the cavity of the uterus to determine the depth of that organ. In fact, he advises strongly against it. By educating the fingers to the sense of touch, the physician can always arrive at a very accurate diagnosis of the size of the uterus by bi-manual palpation alone.

Professor L. Emmett Holt, of this city, thinks an unjust prejudice exists in the minds of physicians in the administration of morphia to children. He says the mistake is made in giving too large a dose at one time. One-eightieth of a grain may be safely given to a child seven or eight months of age, and this dose to be repeated at an interval of a half an hour or so, if no good result has been secured. Small doses, frequently repeated, are, he says, more efficacious and safer than a large dose given at once. He considers this drug as very efficacious in the treatment of certain reflex nervous affections of children, and one that the profession cannot well do without.

Dr. Peter McCahay, of Philadelphia, recently exhibited before the Section of Obstetrics and Gynecology of the Academy of Medicine of this city an instrument which he styles an Atmospheric Tractor. The instrument consists of a disc of soft rubber, about three inches in diameter, and with a soft knob on the outside by means of which it can be manipulated. He introduced it to the section, claiming for it superiority over the forceps in the extraction of the head of the child. It is pressed against the head of the child until the entire air is forced out and the cup adheres closely to the scalp. The advantages claimed for this instrument are its easy mode of application and its absolute freedom from danger to both mother and child. This method is applicable in all forms of labor, as it hastens normal delivery, and in many cases of tedious labor, which might last for hours The employment of this tractor has hastened the process very materially. The inventor has used it a great many times, and has never seen any bad results, either to mother or child, follow its employment. Rupture of the perineum had never taken place in his experience, and this was wholly due to the fact that the pressure of the child's head was not permitted to remain for a long period of time on these parts.

The apparatus might be applied at any stage of labor, provided the os is dilated sufficiently to admit the introduction of the instrument in any portion of the canal. Another advantage it possesses over the forceps is that its manipulation requires but the use of one hand, while the other hand is perfectly free to give further assistance.

An iniquitous institution over in Jersey City, known by the name of the New Jersey Medical and Surgical College, has met with a deserved death at the hands of the Legislature of that State. The alleged professors of this so called institution held their seances in the garret of an old tenement house in Jersey City, and ground out doctors at ten dollars a head. Tinkers tailors, barbers and shoemakers crossed over the ferry from New York, paid the ferry-master six cents both ways, and returned in the next boat with a brand new diploma—of course minus $10, its actual market value—and put up their shingles in New York as full-fledged doctors from the Medical and Surgical College (?) of New Jersey. And who do you think was the president of this noble insittution? An ignorant and arrant quack, whose advertisement helps to swell the so-called medical pages of the morning and evening papers of this city. We are in truth a long-suffering and patient people.

